# Unraveling the Significance of Nanog in the Generation of Embryonic Stem-like Cells from Spermatogonia Stem Cells: A Combined In Silico Analysis and In Vitro Experimental Approach

**DOI:** 10.3390/ijms25094833

**Published:** 2024-04-29

**Authors:** Nima Ghasemi, Hossein Azizi, Thomas Skutella

**Affiliations:** 1 Faculty of Biotechnology, Amol University of Special Modern Technologies, Amol 49767, Iran; nima.ghasemi@ausmt.ac.ir; 2 Institute for Anatomy and Cell Biology, Medical Faculty, University of Heidelberg, Im Neuenheimer Feld 307, 69120 Heidelberg, Germany; thomas.skutella@uni-heidelberg.de

**Keywords:** Nanog, ES-like cells, spermatogonia stem cells, pluripotency, PPI networks, immunostaining

## Abstract

Embryonic stem-like cells (ES-like cells) are promising for medical research and clinical applications. Traditional methods involve “Yamanaka” transcription (OSKM) to derive these cells from somatic cells in vitro. Recently, a novel approach has emerged, obtaining ES-like cells from spermatogonia stem cells (SSCs) in a time-related process without adding artificial additives to cell cultures, like transcription factors or small molecules such as pten or p53 inhibitors. This study aims to investigate the role of the Nanog in the conversion of SSCs to pluripotent stem cells through both in silico analysis and in vitro experiments. We used bioinformatic methods and microarray data to find significant genes connected to this derivation path, to construct PPI networks, using enrichment analysis, and to construct miRNA-lncRNA networks, as well as in vitro experiments, immunostaining, and Fluidigm qPCR analysis to connect the dots of Nanog significance. We concluded that *Nanog* is one of the most crucial differentially expressed genes during SSC conversion, collaborating with critical regulators such as *Sox2*, *Dazl*, *Pou5f1*, *Dnmt3*, and *Cdh1*. This intricate protein network positions Nanog as a pivotal factor in pathway enrichment for generating ES-like cells, including Wnt signaling, focal adhesion, and PI3K-Akt-mTOR signaling. *Nanog* expression is presumed to play a vital role in deriving ES-like cells from SSCs in vitro. Finding its pivotal role in this path illuminates future research and clinical applications.

## 1. Introduction

The generation of ES-like cells—induced pluripotent stem cells (iPSCs) and embryonic stem-like (ES-like) cells derived from germ cells—is a new source of pluripotent cells that has similar functional characteristics to ES cells. In general, iPSCs are generated by the transduction of transcription factors such as Oct3/4, Sox2, Klf4, and c-Myc [1]. In the past few years, several groups have derived ES-like cells from spermatogonia stem cells (SSCs) in vitro [1,2,3]. Gene transcription alternatives are expected in this path, and several papers report changes in the regulation of *Pou5F1*, *Nanog*, *Sox2*, *Dazl*, and *c-Myc* [1,2,4,5]. *Nanog* is one important transcription factor in this network. It has a complicated regulation, is involved in cell fate regulation and the maintenance of pluripotency, and prevents differentiation [6]. Scientists discovered a gene named ENK (early embryo-specific NK) in mouse embryonic stem cells (ES cells) by PCR. This gene, later renamed to *Nanog* by other researchers, is essential for maintaining the undifferentiated state of ES cells. The expression of Nanog is first detected in the early stages of embryo development, specifically in the interior cells of compacted morulae [7]. Murine (m) Nanog is a 280-amino-acid protein with three domains. It has a serine-rich N-terminal, NK-2 type homeodomain, and a highly conserved tryptophan-rich C-terminal domain. Both N-terminal and C-terminal domains have transcriptional activity when fused to the Gal4 DNS-binding domain, but the C-terminal domain’s activity is at least seven times more than the N-terminal domain’s activity [7]. Human (h) and murine (m) Nanog proteins exhibit a high degree of amino acid sequence similarity (about 58%) and shared structural organization [8]. Nanog is not much expressed in human tissues, and its gene becomes partially silent after birth. Nanog is expressed in stem cells and contributes to proliferation, apoptosis, and cell fate, and in ES cells, it is involved in pluripotency maintenance [6]. Nanog is expressed in pluripotent genes and gradually downregulates during differentiation. Based on previous research, it has been shown that an Oct4/Sox2 motif on the upstream region of the 5′ promoter is essential for regulating Nanog activity. Reports demonstrate that FoxD3 can be considered a Nanog activator alongside Oct4 and Sox2, while TCF3 and p53 can negatively regulate Nanog by binding to its promoter region [9,10]. Additionally, SNAIL and BM-1 have a positive effect on Nanog expression [11,12]. Further experiments demonstrated that Oct4 and Sox2 proteins are important for Nanog function in vitro and in vivo [13]. Nanog expression is regulated by several factors such as miRNAs, lncRNAs, DNA methylation, transcription factors, and protein regulators. Nanog overexpression alone in spermatogonia stem cells cannot lead to the generation of pluripotent cells [14]; during the conversion of SSCs to ES-like cells, Nanog is significantly upregulated, and it is considered one of the key regulators in this pathway [2,4,5]. 

Based on our experiments, *Nanog* is highly differentially expressed between SSC and ES-like cells. According to in silico analysis and PPI networks, *Nanog* is among other pluripotency genes and has a strong relationship with them based on network parameters. Enrichment analysis showed that Nanog plays a significant role in several cellular pathways, such as regulating gene expression, cell differentiation, and cell proliferation. We used in vitro experiments to test and confirm our in silico results. A Fluidigm qPCR test between the ES-like and SSC populations has also shown a high mRNA expression of Nanog in ES-like cells. The immunocytochemical staining test showed a higher expression of Nanog in the ES-like cell population compared with the SSC population. 

This study aims to elucidate Nanog’s intricate interactions among genes, protein networks, and their biological roles, laying the foundation for prospective experiments analyzing the switch from SSCs to ES-like cells. This knowledge has promising implications for stem cell biology and regenerative medicine.

## 2. Results

### 2.1. Microarray Analysis and Identification of Differentially Expressed Genes (DEGs) in the Derivation of ES-like Cells from SSCs

**Figure 1:**
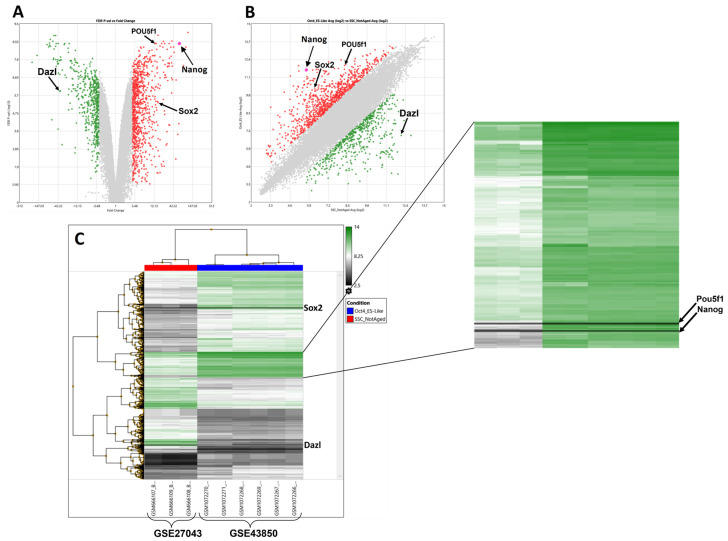
Results of microarray analysis using TAC (v.4.0). Nanog is shown with a black arrow (**A**) Volcano plot of DEGs based on FDR *p*-value vs. fold change. (**B**) Scatter plot of DEG fold change in two compared groups. (**C**) Heat map of 1615 DEGs.

In the first step, microarray dataset analysis was performed to identify all significant DEGs in this derivation path using TAC (v.4.0) between ES-like cells and SSCs. All data were normalized and principal component analysis (PCA) was conducted, revealing that 97.8% of the variance is explained by the principal components (Supplementary Figure S1). Among the 28,944 genes, 1615 DEGs were identified between samples. A total of 1029 genes were upregulated, and 586 genes were downregulated in ES-like cells compared to SSCs (Figure 1). *Nanog* was among the top 100 differentially expressed genes based on an FDR *p*-value with 61.95 fold changes and a 3.54 × 10^−9^ FDR *p*-value with other critical genes such as *POU5f1* with a 3.54 × 10^−9^ FDR *p*-value and 12.8 fold changes, *Sox2* with a 4.92 × 10^−6^ FDR *p*-value and 15.67 fold changes, *Dazl* with a 1.21 × 10^−6^ FDR *p*-value and −37.94 fold changes, and *N-Myc* with a 0.0001 FDR *p*-value and 4.78 fold changes. Further analysis was carried out using different comparison layouts on additional ES-like and ES samples within GSE43850 to provide additional confirmation of our main analysis. All these various combinations validated our results to some extent (Supplementary Figure S2).

### 2.2. Protein–Protein Interaction (PPI) Networks

**Figure 2:**
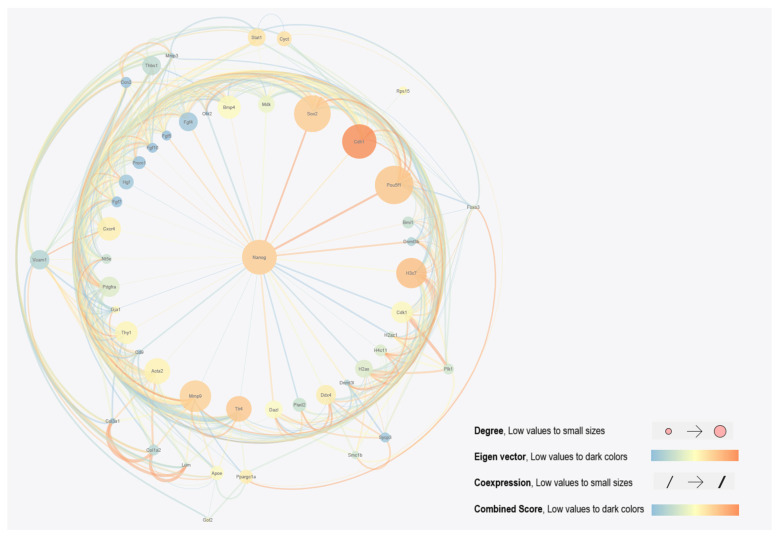
PPI network of 50 hub genes generated in Cytoscape. Hub genes are filtered based on network parameters. Nodes and edges are visualized based on parameter weights such as degree, eigenvector, Co-expression, and combined score. Cdh1, Nanog, Sox2, Pou5f1, and H3c7 are among network-critical proteins based on degree and eigenvector.

**Figure 3:**
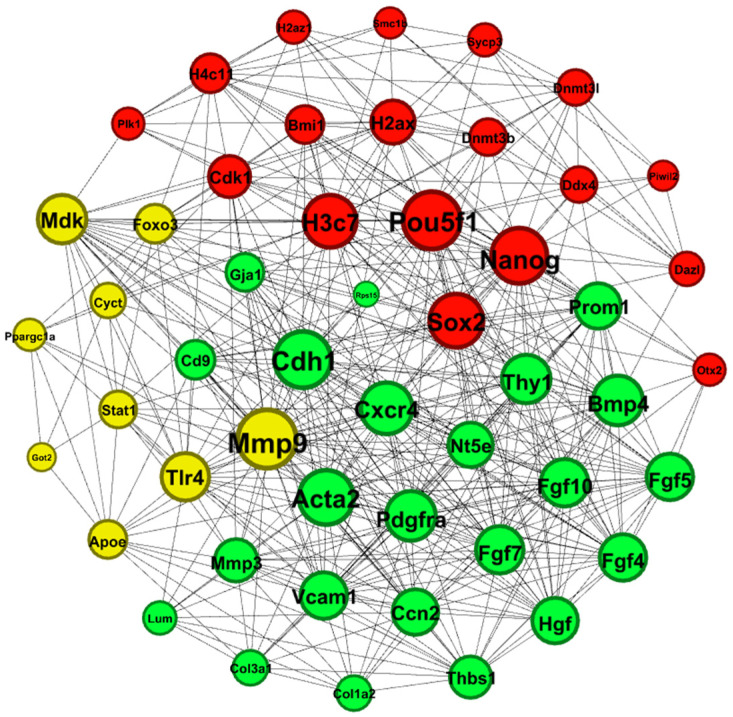
PPI network of three clusters. A total of 50 hub genes under modularity algorithm and clustering filters were specified by Gephi. Larger nodes have more connected nodes and larger degrees. Pou5f1, Nanog, Sox2, H3c7, Cdh1, Mmp9, and Cxcr4 are important proteins in the network and it is predicted that their presence is vital for the network to drive its related pathways.

We enter the number of 1615 DEGs filtered in the last step to the STRING database to construct the PPI network of these given genes. This constructed network is then imported into the Cytoscape app (v. 3.6.0). Different parameters within the network can show a node’s importance. Centrality is the property of a node. The more connections a node has and the more effective those connections are, the higher its centrality. There are several different ways to calculate centrality in a network, each emphasizing different aspects of a node’s importance, degree, betweenness, closeness, and eigenvector. Degree centrality is the property of a node and shows the number of connected nodes to it. Nodes with a higher degree of centrality are considered to be more important because they have a wider reach and can potentially influence a larger portion of the network. Closeness centrality calculates the average shortest path length between a node and all other nodes in the network. Betweenness centrality considers how often a node lies on the shortest path between two other nodes. The higher the betweenness centrality, the more central it is because it acts as a bridge between different parts of the network and potentially controls information flow. The eigenvector is a more complex method and considers the importance of a node based on the “importance” of its neighbors. Nodes connected to other central nodes are themselves considered more central. This method is useful in identifying hubs or influential players within a network. To obtain more relevant DEGs and a more accurate network, we need to filter DEGs. First, we calculate the network parameters degree, betweenness centrality, and closeness centrality with the built-in algorithm and eigenvector parameter by Centiscape app (v.2.2). After several attempts of trial and error, we assume that the calculated network parameters are optimized (Supplementary Table S1). A network with 50 HUB genes is constructed based on our filtration method (Figure 2). In the network, lower values are represented in blue, and as the values gradually increase, the color transitions to orange. This new network with 50 HUBs is then imported to the STRING database for reconstruction based on Text mining, Experiments, Databases, Co-expression, Neighborhood, Gene Fusion, and Co-occurrence sources. This helps us to have a more accurate and trustworthy network. The reconstructed network is then imported to Gephi (v. 0.10.1) for further analysis. We analyze this imported network using a modularity algorithm to obtain clustering groups. Three clustered groups are highlighted by an identical color (red, yellow, and green) for better identification. Nanog is connected to 17 other genes with critical pluripotency factors, such as Pou5f1 and Sox2, in its cluster (Figure 3). To obtain more network characteristics for better analysis, we use the EPC method of the Cytohubba (v.0.1) plugin to rank genes connected to Nanog in the top 50 hub gene network. For this purpose, we select nodes connected to Nanog and their adjacent edges and then apply the EPC method to them. Based on the EPC method, nodes are colored from highly essential nodes (red) to essential nodes (yellow) based on evolutionary conservation and biological relevance (Supplementary Figure S3). In the EPC network, Nanog, Sox2, Pou5f1, Cdh1, and Mmp9 are the top nodes with the highest EPC score. Nt5e, Prom1, Cxcr4, Mdk, H3c7, and the rest are growth factors, and Fgf, Bmp, Pdgf, and Hgf are also among the highlighted nodes.

### 2.3. Enrichment Analysis

**Figure 4:**
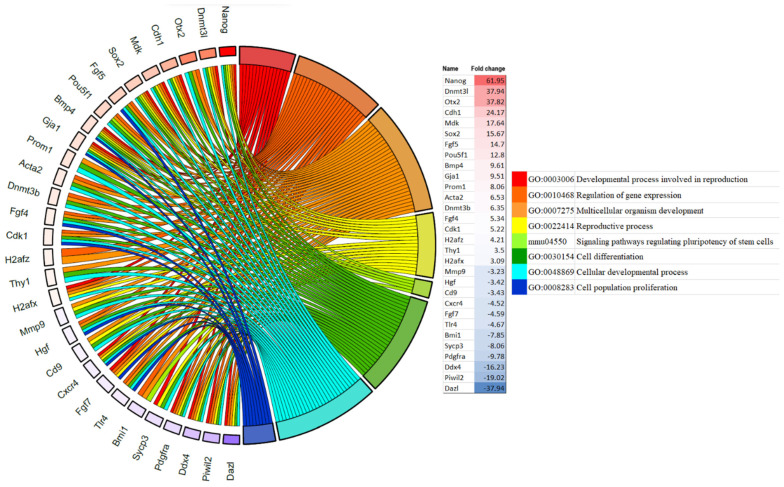
GO chord enrichment plot. Enrichment results of Nanog cluster genes were organized and visualized in a chord plot. Every color represents a certain pathway within the plot. Genes are organized based on their fold change, such that red shows upregulated genes and blue shows downregulated ones. As color fades, fold change decreases.

Two networks were selected for enrichment analysis. First, we apply enrichment analysis on 50 filtered HUB networks to investigate critical enriched pathways in the whole process. For this purpose, data from GO terms, KEGG, TISSUES, and Wikipathways are selected and analyzed. We select the top 10 enriched pathways based on the false discovery rate (FDR). Based on GO results, protein binding, cell adhesion molecule binding, chromosomes, positive regulation of the macromolecule metabolic process, and positive regulation of the cellular process are significantly enriched terms (Supplementary Table S2). TISSUES, KEGG, and Wikipathways showed a significant change in pluripotent stem cells, embryonic cell lines, PI3K-Akt signaling pathways, signaling pathways regulating the pluripotency of stem cells, ESC pluripotency pathways, and focal adhesion terms (Supplementary Table S3). Secondly, we checked the enrichment pathways of the Nanog cluster, covering 17 genes, to dig more into enriched pathways that can play a significant role in our study. The network was imported to Cytoscape by the STRING plugin, and enrichment analysis was applied to them. The STRING app uses a variety of databases such as GO terms, KEGG, and TISSUES to run enrichment analysis. Nanog is predicted to be present in developmental processes involved in reproduction, gene expression regulation, signaling pathways regulating the pluripotency of stem cells, and cell differentiation. In the next step, enrichment results were selected based on relevance and Nanog participation and visualized in a chord plot by the SRplot online platform (https://www.bioinformatics.com.cn/srplot (accessed on 30 December 2023) (Figure 4). 

### 2.4. Protein-LncRNA-miRNA Network

**Figure 5:**
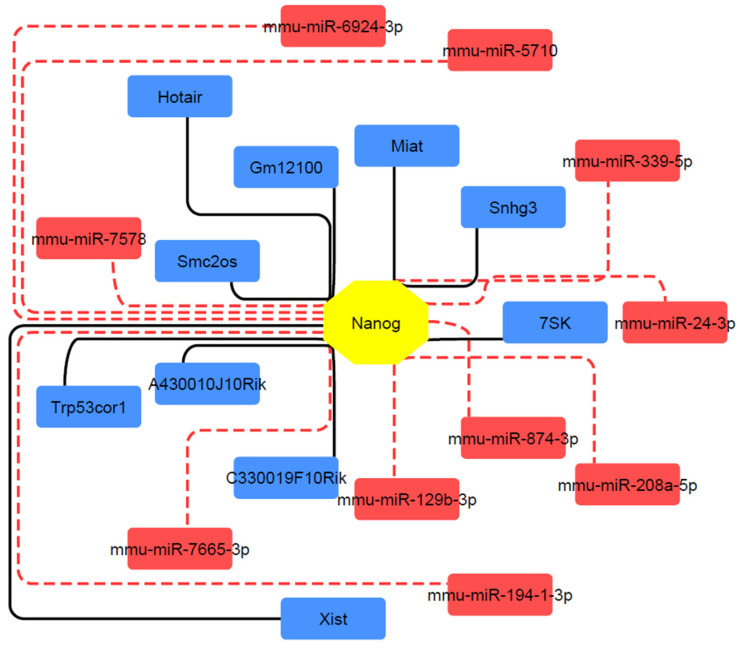
Visualization of protein–miRNA–lncRNA network. Totals of 10 miRNAs and 10 lncRNAs were identified through bioinformatic methods. Red rectangles and their adjacent edges indicate miRNAs. Blue rectangles and their adjacent edges indicate lncRNAs.

Each of these miRNAs or LncRNAs has its specific regulatory role on Nanog, so the construction of Nanog-LncRNA-miRNA can help in identifying and predicting each lncRNA or miRNA that can change the expression pattern of Nanog based on the content of this study. To build the network, the top 10 mutual miRNAs were predicted and selected via mirdb, TargetScan, miRWalk, and RNAInter. The top 10 LncRNAs based on the score were predicted and gathered from RNAInter. These data were imported and visualized in the Cytoscape app (Figure 5).

### 2.5. Fluidigm qPCR Analysis of Nanog Expression in ES-like Cells and SSC Population

**Figure 6:**
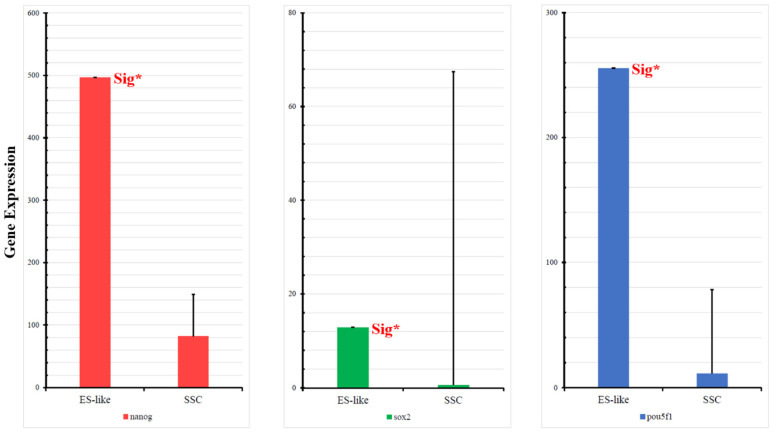
Result of Fluidigm qPCR. Fluidigm qPCR analysis shows that mRNA expression of Nanog, Pou5f1, and Sox2 significantly increases during the transformation of SSCs to ES-like cells. C57 MEF population was used as a reference cell for normalization. The Y-axis represents the fold change in mRNA expression compared to MEF feeder cells. (The “Sig*” sign denotes a significant gene expression value (*p* < 0.05) for each gene in each test).

We used Fluidigm qPCR to quantify the mRNA expression between ES-like cells and SSCs to better understand the Nanog expression rate in vitro. As we predicted via in silico analysis, the ES-like cell population shows a higher expression rate of Nanog mRNA expression than SSC cells (*p* < 0.05, Figure 6). Besides for further confirmation, we applied Fluidigm qPCR for other connected critical genes within the network such as *Sox2* and *Pou5f1*. The difference in the expression pattern of these selected candidates is significant between these two groups and can somewhat validate our in silico analysis.

### 2.6. Nanog Expression Comparison by Immunostaining Method between SSCs and ES-like Cells Population

**Figure 7:**
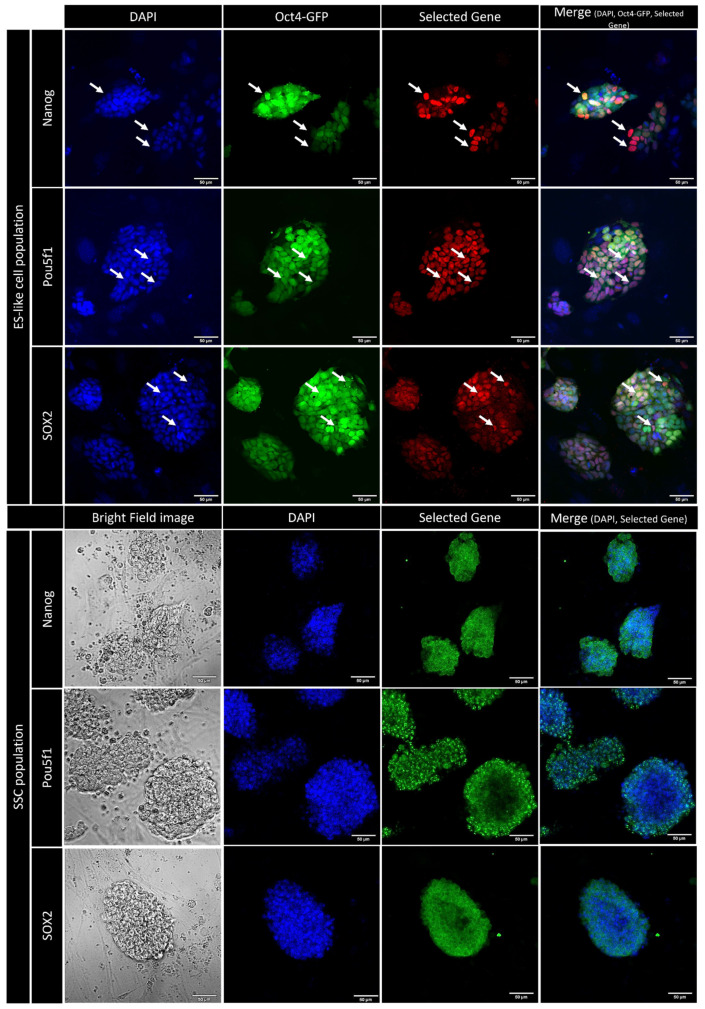
An immunocytochemical (ICC) test of studied cell populations. Immune staining was performed on SSCs and ES-like cells on genes, Nanog, Oct4-ab (POU5f1), and SOX2 that were selected based on study design. In the ES-like cell population, blue represents DAPI staining, green represents Oct4-GFP marker, and red represents selected gene. In the SSC population, blue represents DAPI and green represents selected gene (scale bar = 50 µm).

An immunocytochemical (ICC) test was performed between two cell populations, SSCs and ES-like, to determine the Nanog protein concentration difference (Figure 7). Immunocytochemical images were obtained from the confocal canning UV-laser microscope, and these images show a higher expression of Nanog in ES-like cells than in SSCs. For further confirmation and better bridging between in silico analysis and in vitro experiments, we performed immunocytochemical staining for Sox2 and Pou5f1. Our results show a higher expression of *Sox2* and *Pou5f1* genes in the ES-like population than in SSCs. This result was expected due to the close relationship of Nanog, Pou5f1, and Sox2 in the PPI network and enrichment analysis.

## 3. Discussion

In silico and microarray analysis showed that many genes are involved in producing ES-like cells from SSCs in vitro. These two cellular populations show different expression patterns. Genes such as *Tdgf1*, *Nanog*, *Pou5f1*, *Apela*, *Sox2*, and *Cdh1* are among the important upregulated genes, and *Xlr5a*, *Tex11*, *Nxf2*, and *Dazl* are among the most significant differentially expressed genes in this path. Nanog has both a high fold change and a low FDR *p*-value. So, it can play a significant role in this pathway and is highly connected to important pluripotency genes. Boyer et al. concluded that Oct4, Sox2, and Nanog are master pluripotency transcription factors (TFs). As concluded in 2023, Pou5f1 is essential for pluripotency maintenance in the derivation path of SSCs to ES-like cells and gradually decreases after differentiation stages [15]. In addition, they demonstrated that *Tdgf1* is among the top genes with a high expression in ES cells [16]. Chen et al. reported that Dazl inhibits Sox2, Sall4, and Suz12 and, as a result, limits pluripotency [17]. Dazl downregulation in this derivation path can stimulate pluripotency and the derivation of pluripotent cells. As can be seen in the network, Nanog has a robust association with Cdh1. Hawkins et al. reported that E-cadherin has a significant role in Nanog expression maintenance [18]. Basira Najafzadeh et al. also reported a relationship between Nanog and E-cadherin in cancer stem cell development [10]. As Nanog connects with DNA methylation proteins such as Dnmt3 in the network, it can be concluded that Nanog’s connection with these proteins is important in deriving ES-like cells from SSCs. Siba Shanak et al. concluded that pluripotency factors such as Nanog and DNA methyltransferases are present in the pluripotency path and can regulate each other’s expression [19]. Nanog has a strong connection with Sox2 and Pou5f1 in the PPI network, and it can be concluded that Nanog performs its action in this pluripotency path by cooperating with these proteins. As David J. Rodda et al. concluded, there is a close link between these three gene promoters. They reported that the Nanog promoter is influenced by Sox2 and Pou5f1, which is essential for maintaining pluripotency and self-renewal [13]. The Wikipathways enrichment analysis showed a significant change in “mechanisms associated with pluripotency”, “focal adhesion: PI3K-Akt-mTOR signaling pathway”, “ESC pluripotency pathways”, and “miRNAs and TFs in iPS Cell Generation”. KEGG indicates a considerable change in the “PI3K-Akt signaling pathway”, “Rap1 signaling pathway”, and “signaling pathways regulating pluripotency of stem cells”. Yoon et al. reported in 2021 that the PI3K/Akt pathway takes center stage, demonstrating its role in regulating the expression of Nanog in sarcoma spheroid-forming cells. Furthermore, it actively promotes cancer stem cell phenotypes, including the formation of spheroids and resistance to therapeutic interventions [20]. Blinka et al. pointed out Nanog’s critical role in ESC pluripotency pathways, and Nanog expression is essential for both the initiation and ongoing maintenance of pluripotency [21]. Based on Li Li et al. [22], Rap1, while not directly influencing the expression of Nanog, holds a crucial role in the endocytic recycling pathway. This process is vital for the creation and upkeep of E-cadherin-mediated cell–cell cohesion, which, in turn, is crucial for forming colonies and the self-renewal of hESCs. In addition to the discussed connections, to the best of our knowledge, proteins such as Smc1b and Sycp3 have not been previously associated with ES-like derivation from SSCs in the literature. Based on our enrichment analysis in silico, Smc1b contributes to functions related to nucleic acid binding, the synaptonemal complex, and sister chromatid segregation. Similarly, Sycp3 is involved in DNA metabolic processes, Meiosis I and II, synaptonemal complex organization, and chromatin organization. The association of Nanog with its connected proteins in the PPI network cluster represents another aspect that requires further fundamental studies to enhance our understanding of the underlying mechanisms within this pathway. Nanog forms a cluster with other genes within the network. In addition to being connected with Sox2 and Pou5f1, Nanog is in the same cluster with them. So, it is highly predicted to be among the key regulators that induce the derivation of pluripotent cells from SSCs. This protein cluster is predicted to be present in important pathways such as “cell differentiation”, “signaling pathways regulating pluripotency of stem cells”, and “regulation of gene expression”. We suggest from this derivation path that Nanog upregulation is one of the crucial elements.

Methylation profiles also play a crucial role in pluripotency. It is predicted that the methylation status of involved genes in the derivation of ES-like cells from SSCs significantly changes. Research indicates that derived ES-like cells from SSCs show an increase in demethylation for Nanog, H19, and Pou5f1 [1]. According to methylation studies, the hypomethylation of Pou5f1 and Nanog was evident in ES-like cells obtained from SSCs [23]. The Transcriptional Regulatory Network (TRN) analysis revealed that PRMT1 and PRMT8, which are involved in arginine methylation processes, are under the regulation of ZFP143 and Nanog. PRMT1 and PRMT8 are involved in pluripotency. These data suggest that arginine methylation driven by Nanog might play a significant role in acquiring pluripotency during the SSC to mSSC (multipotent spermatogonia stem cells) reprogramming [24].

In addition, miRNA and lncRNA interactions are effective on Nanog status. This regulatory protein–miRNA–lncRNA is considerable and discussed by related studies. Our bioinformatic analysis reveals miRNAs such as miR-5710, miR-339-5p, miR-24-3p, miR129b-3p, and miR7578. Lee et al. characterize miR-24-3p as a microRNA with anti-pluripotent properties. The targeting sites of miR-24-3p are situated in the 3′UTRs of Nanog [25]. To our knowledge, no references to other predicted microRNAs have been identified. However, additional research and analysis are imperative to explore this aspect further. Furthermore, the bioinformatic analysis predicted lncRNAs like Hotair, Miat, Xist, and Snhg3. Wang et al. investigated the involvement of Hotair in the stem cell signaling pathway by assessing the levels of Nanog following the knockdown of Hotair. They concluded that Hotair reduction did not significantly impact the expression of Nanog, a finding further validated by examining protein levels [26]. Wang et al. mentioned that Nanog and activation, facilitated by the high expression of lncRNA Xist, play a pivotal role in cancer immunity and brain metastasis in triple-negative breast cancer (TNBC) cells. They concluded that this occurs by stimulating the PI3K/AKT/mTOR signaling pathway [27]. Lu et al. showed the crucial role of Snhg3 in sustaining self-renewal and pluripotency in murine ESCs. In mESCs, Nanog acts as the primary regulator for Snhg3, and reducing Nanog expression significantly lowered Snhg3 levels. Conversely, knocking down Snhg3 led to a notable decrease in Nanog expression. The depletion of Snhg3 blocked the development of early mouse embryos, compromised the self-renewal ability of mESCs, and caused alterations in pluripotency. On the other hand, overexpressing Snhg3 promoted self-renewal and suppressed the differentiation of mESCs [28].

The findings from the Fluidigm analysis provide additional support to the in silico analysis. The Fluidigm analysis gives a proper vision to the mRNA levels of our cell population. The graphs generated demonstrate a significant rise in Nanog expression within neonate ES-like cells compared to neonate SSCs. Similar to our experimental results, Asadi et al. [29] reported a rise in Nanog expression in ES-like cells compared to SSCs in the derivation path by the qPCR method. Similarly, Azizi et al. showed a significant upregulation of Nanog, Sox2, and Pou5f1 in transforming SSCs to ES-like cells in an age-dependent manner [2,4]. Immunocytochemical staining was conducted on both the SSC and ES-like cell populations, aiming to delve more deeply into the intracellular levels of critical proteins within the protein–protein interaction (PPI) network, specifically emphasizing Nanog. Similar to microarray analysis that showed a significant upregulation of Nanog in transforming ES-like cells from SSCs, the results of immunocytochemical staining on SSCs indicate a relatively low Nanog concentration within the cells. Conversely, the immune staining of ES-like cells reveals a significantly elevated concentration of Nanog within the cells. Moreover, the comparison of Sox2 and Pou5f1 concentrations between ES-like cells and SSCs demonstrates a substantial disparity, with ES-like cells exhibiting a notably higher concentration of these proteins than SSCs. This in vitro result can validate our microarray results, which showed a positive fold change of Sox2 and Pou5f1. These findings not only provide valuable insights into the nuanced protein expression profiles but also serve as supportive evidence for our in silico analysis, wherein Nanog emerged as a pivotal and differentially expressed gene during the transformation of SSCs into ES-like cells. Previous studies reported that there is a higher concentration of Nanog and other pluripotent factors in ES-like cells than SSCs [29,30,31]

The expression status of the Nanog gene among ES-like cells derived from SSCs has been assessed in previous studies with a comparative perspective. As far as we know, most but not all of these studies have reported a higher Nanog gene expression or protein concentration by in vitro experiments. Still, comprehensive investigations into Nanog properties along this pathway are lacking. In this study, we employed both in silico analysis and in vitro experiments to mutually reinforce each other, aiming for more robust results. Microarray analysis identified Nanog as a crucially differentially expressed gene in this path. Its interactions with other pivotal proteins in the PPI network and its presence in enriched pathways underscore Nanog’s role as a key regulator in our studied biological phenomenon. Fluidigm qPCR analysis revealed an elevated mRNA level of Nanog in the ES-like cell population, and correspondingly, immunocytochemical staining demonstrated a higher concentration of Nanog protein in ES-like cells. These two in vitro experiments complement each other, confirming Nanog’s status in vitro. Utilizing these in vitro experiments to complement and validate our in silico analysis enhances the comprehensiveness of our study. This synergy allows our computational analysis to transcend mere predictions.

## 4. Material and Methods

### 4.1. Microarray Data Analysis and Data Normalization

Two independent gene expression datasets were used in this research. GSE43850 consisted of 46 samples and GSE27043 consisted of 8 samples, downloaded from https://www.ncbi.nlm.nih.gov/geo/ (accessed on 16 November 2023). We selected six ES-like cell samples (Oct4mGS) from GSE43850 [32] and three SSC samples (SSC_Not aged) from GSE27043 [33] for our differentially expressed gene analysis. All samples were profiled using the GPL624 (MoGene-1_0-st) platform. We used TAC (v.4.0) to analyze CEL files. All samples normalized by the RMA method and ebayes ANOVA method were selected. The gene level *p*-value was <0.05 and the gene-level fold change was <−2 or >2.

### 4.2. Protein–Protein Interaction (PPI) Network Construction and Modularity Analysis

STRING (v.12.0) was used to predict the protein–protein interaction between given DEGs (https://string-db.org/ (accessed on 17 November 2023). The organism was set to Mus musculus with a medium confidence score (0.400) and the sources of prediction were set to Text mining, Experiments, Databases, Co-expression, Neighborhood, Gene Fusion, and Co-occurrence. The given PPI network was then imported to Cytoscape (v.3.6.0) for further filtration and analysis. We used Centiscape (v.2.2), a Cytoscape plugin, to calculate eigenvector network parameters. Protein functional clusters were calculated by the Gephi app (v. 0.10.1) via the modularity built-in function. Cytohubba (v.0.1) is a Cytoscape plugin that calculates the importance of every node in the network based on several algorithms like Edge Percolated Component (EPC), Maximum Neighborhood Component (MNC), Density of Maximum Neighborhood Component (DMNC), Bottleneck, EcCentricity, and closeness. We used the EPC method in our experiment to rank nodes of the provided network.

### 4.3. Enrichment Analysis

For a better understanding of DEG’s role in the network and to seek functional roles of important nodes of the network, we used enrichment analysis using the enrichment analysis tool of the STRING app in Cytoscape. Enrichment results were gathered from KEGG, TISSUES databases, and GO terms. The SRplot online platform (https://www.bioinformatics.com.cn/srplot (accessed on 30 December 2023) was used for enrichment result visualization. 

### 4.4. Protein–LncRNA–miRNA Regulatory Network Related to Nanog Construction

miRNA data for predicting the impact of miRNA networks on the Nanog gene were collected from miRDB (https://mirdb.org/mirdb (accessed on 17 November 2023), TargetScan (https://www.targetscan.org/ (accessed on 17 November 2023), and miRWalk (http://mirwalk.umm.uni-heidelberg.de/ (accessed on 17 November 2023). Individuals common to these three datasets were selected, and the network was formed using Cytoscape software (v.3.6.0). For lncRNA data, we used RNAInter (http://www.rnainter.org/ (accessed on 17 November 2023), and the first 10 lncRNAs based on score were selected and added to the network.

### 4.5. Isolation of Spermatogonia Stem Cells

In the present study, animal experiments received approval from the Institutional Animal Care and Ethics Committee of Amol University of Special Modern Technologies. All aspects of animal care adhered to the guidelines set forth by Amol University of Special Modern Technologies in Amol, Iran. Testicular cells were extracted from Oct4-promoter reporter GFP transgenic mice of C57BL/6 and 129/Sv strains, aged 6 days to 6 months, following decapsulation and treatment using a one-step enzymatic digestion method. Following the removal of the tunica albuginea, the dissociated testicular tissue was exposed to a digestion solution at 37 °C for 8 min. The enzymatic digestion was halted by adding 10% ES cell-qualified FBS and gently pipetting to achieve a single-cell suspension. After centrifugation, the samples were rinsed with DMEM/F12, strained through a 70 μm cell strainer, and centrifuged again for 10 min at 1500 rpm. The supernatant was discarded, and the suspension of testicular cells was seeded onto culture dishes coated with 0.2% gelatine [2,15].

### 4.6. Culture of Spermatogonia Stem Cells

Based on our previous study [2], the suspension of adult testicular cells was cultured by plating them onto 0.2% gelatin-coated plates in an SSC (Spermatogonial Stem Cell) culture medium. This medium consisted of StemPro-34 medium, 5 μg/mL bovine serum albumin (Sigma Aldrich, St. Louis, MO, USA), 1% L-glutamine (PAA, Whatman, MA, USA), 0.1% ß-mercaptoethanol (Invitrogen, Waltham, MA, USA), 6 mg/mL D+ glucose (Sigma Aldrich, St. Louis, MO, USA), 1% nonessential amino acids (PAA, Whatman, MA, USA), 1% N2-supplement (Invitrogen, Waltham, MA, USA), 1% penicillin/streptomycin (PAA, USA), 1% MEM vitamins (PAA, USA), 60 ng/mL progesterone (Sigma Aldrich, St. Louis, MO, USA), 10 ng/mL FGF (Sigma Aldrich, St. Louis, MO, USA), 20 ng/mL epidermal growth factor (EGF), 100 μg/mL ascorbic acid (Sigma Aldrich, St. Louis, MO, USA), 30 ng/mL estradiol (Sigma Aldrich, St. Louis, MO, USA), 30 μg/mL pyruvic acid (Sigma Aldrich, St. Louis, MO, USA), 8 ng/mL GDNF (Sigma Aldrich, St. Louis, MO, USA), 100 U/mL human LIF (Millipore, Burlington, MA, USA), 1% ES cell qualified FBS, and 1 μL/mL DL-lactic acid (Sigma Aldrich, St. Louis, MO, USA). This culture was maintained at 37 °C with 5% CO_2_ in the air.

### 4.7. Generation and Culture of ES-like Cells Derived from Spermatogonia Stem Cells

As H Azizi et al. mentioned earlier, ES-like cells can be derived from SSCs in a culture medium [4]. Based on our earlier study, Transgenic mice with a GFP reporter under the control of the *OCT4* promoter, derived from the C57BL/6 adult mouse strains, were cultured in a medium designed for mouse spermatogonia stem cells. Approximately six weeks (41–125 days) after the initiation of the culture, ES-like cells expressing a substantial level of OCT4-GFP were successfully generated. After subjecting the colonies resembling embryonic stem cells to trypsinization in a mouse ES medium, individual cells were isolated. This medium consisted of KO-DMEM (or high-glucose DMEM), FBS, MEM NEAA solution, L-glutamine, Pen-Strep, mercaptoethanol, and LIF, and was cultivated on a layer of MEF feeder cells. The colonies exhibiting ES-like characteristics were cultured in mESCs media and underwent passage every 3–4 days. Following this, the resultant ES-like cells were cultured in mESC medium, achieving confluence within approximately 4–5 days of culture initiation. Upon reaching confluence, cells were transferred onto fresh MEF feeder layers after rinsing with PBS and treating with trypsin–EDTA for 3 min. The trypsin–EDTA was then inactivated using 15% FBS [2].

### 4.8. Fluidigm qPCR Gene Expression Analysis

The quantification of *Nanog* gene expression in SSCs, ES-like cells, and Mouse Embryonic Fibroblasts (MEFs, used as a control) was conducted using Fluidigm Dynamic Array Chips. RNA extraction for Fluidigm qPCR was conducted on the SSC population after the 3rd–4th culture, and similarly, RNA extraction for Fluidigm qPCR was performed on the ES-like cell population after the 3rd–4th culture as well. Glyceraldehyde-3-phosphate dehydrogenase served as the housekeeping gene for normalization. Cell selection was performed using a micromanipulator, followed by lysis with a solution comprising 1.3 μL TE buffer, 9 μL RT-PreAmp Master Mix, 0.2 μL R.T./Taq Superscript III (Invitrogen, USA), 2.5 μL 0.2× assay pool, and 5.0 μL Cells Direct 2× Reaction Mix (Invitrogen, USA). mRNA underwent reverse transcription into cDNA through a process utilizing a reverse transcriptase enzyme. This cDNA, specific to the sequence, underwent preamplification in a single tube. The targeted transcripts’ quantity was determined using TaqMan qPCR on the BioMark Real-Time quantitative PCR (qPCR) system. Reverse transcription took place at 50 °C for 15 min using the reverse transcriptase enzyme, followed by inactivation through heating to 95 °C for 2 min. The denaturation of cDNA occurred at 95 °C for 15 s. Subsequently, products were preamplified at 60 °C for 4 min across 14 cycles. The preamplified products underwent up to a 5-fold dilution before analysis using Universal PCR Master Mix and TaqMan gene expression assays (ABI) in 96.96 Dynamic Arrays on a BioMark System. Each sample underwent analysis in two technical replicates. For data analysis, missing data on the Biomark system were substituted with a Ct value of 30, and normalization was conducted using GAPDH. Each sample was analyzed in three replicates. The expression fold change of mRNA compared to MEF feeder cells was determined. Analysis was performed using GenEx software (v. 7.0), Excel, and SPSS (v. 27.0.10.0) [2].

### 4.9. Immunocytochemical Staining

Testicular cells were first fixed rigorously with a potent 4% paraformaldehyde solution and subsequently subjected to a thorough permeable staining procedure utilizing a robust 0.1% Triton/PBS solution. Following this, the cells underwent a stringent blocking phase employing a formidable 1% BSA/PBS solution. The subsequent treatment involved the application of commanding primary antibodies targeting OCT4, SOX2, and NANOG. Notably, this study harnessed the formidable Anti-OCT4 antibody (ab200834) to discern OCT4 expression, the potent Anti-Nanog antibody (ab109250) for NANOG, and the robust Anti-SOX2 antibody (ab92494) for SOX2. These antibodies were employed with unwavering precision in both immunohistochemistry and immunocytochemistry. Subsequently, secondary antibodies tailored for specific fluorochrome species were deployed during incubation, and the marked cells were intensively stained with a concentration of 0.2 g/mL of the potent 4′,6-diamidino-2-phenylindole (DAPI) dye, serving as a potent agent for nuclear counterstaining. The examination of positively labeled testicular cells was carried out with great precision using cutting-edge confocal microscopy and a state-of-the-art Zeiss LSM-TPMT camera from Oberkochen, Germany [2].

### 4.10. AI Tools

The utilization of artificial intelligence tools for the generation of data, the composition of article sections, the formulation of scientific conclusions, and any other applications that might potentially result in scientific misrepresentation was expressly prohibited. These tools were strictly employed for editorial correction and grammar checks within the text. Their usage was confined to refining and enhancing the clarity and accuracy of the content without engaging in any form of data manipulation or misrepresentation in the scientific discourse.

### 4.11. Statistical Analysis

The experiments were iterated a minimum of three times. The data underwent statistical analysis using Statistical Package for the Social Sciences (SPSS) version 27.0. Following an assessment of the Shapiro–Wilk normality test results for the data derived from the Fluidigm test, it was determined that the gene expression data did not follow a normal distribution. Consequently, non-parametric tests such as the Kruskal–Wallis H test were employed, and then a one-way analysis of variance (one-way ANOVA) test was proceeded to assess the significance, as well as the Bonferroni test. Statistical reliability of the variation between groups was considered when a *p*-value < 0.05 was obtained.

## 5. Conclusions and Future Insights

ES-like cells can play a significant role in various research fields and clinical applications. Several crucial genes exhibit different expression patterns during the conversion of SSCs to ES-like cells, including *Cdh1*, *Dazl*, *Nanog*, *Pou5f1*, *Sox2*, *Dnmt3*, *H3c7*, *Fgf*, and *Ccn2*, as determined by in silico analysis. Additionally, miRNAs such as miR-339-5p, miR-129b-3p, and miR-194-1-3p, along with lncRNAs like 7SK and Xist, contribute to the regulation of target genes within the network. Studying each of these genes gives us more understanding of ES-like cell states. *Nanog* is known as one of the important pluripotent genes. *Nanog* is among the upregulated genes in this path. It interacts with important genes to enrich several pathways to help this derivation pathway. Our examination of Nanog represents a foundational analysis of its condition and offers predictions regarding its roles and functions in transformed cells, paving the way for future studies. Much remains to be understood, and further applied research concerning Nanog and its significance is essential in this field of study. Identifying more reliable and efficient methods for obtaining superior ES-like cells from SSCs, particularly for research and medical applications, is a promising avenue for exploration. 

## Data Availability

All data generated or analyzed during this study are comprehensively presented in this article. For additional inquiries, please direct them to the corresponding author.

## Supplementary Materials

The following supporting information can be downloaded at https://www.mdpi.com/article/10.3390/ijms25094833/s1.
