# Supplementary material for: Unraveling the Significance of Nanog in the Generation of Embryonic Stem-like Cells from Spermatogonia Stem Cells: A Combined In Silico Analysis and In Vitro Experimental Approach
Source: Int J Mol Sci. 2024 Apr 29;25(9):4833. doi: 10.3390/ijms25094833 (PMC11084519; doi:10.3390/ijms25094833)
Supplement: Supplementary file 1 [file ijms-25-04833-s001.zip › ijms-2907549-supplementary.pdf]

Supplementary Tables and Figures

Supp. Table S1*. Optimized network parameters to obtain HUB network*

Column1	Network parameter	Range
**1**	Degree	Between 30 and 93 inclusive
**2**	Betweenness Centrality	Between 0.003 and 1 inclusive
**3**	Closeness Centrality	Between 0.3 and 1 inclusive
**4**	Eigenvector	Between 0.009 and 0.219 inclusive

Supp. Table S2*. Top 10 enriched pathways of Gene Ontology terms based on False discovery rate. Nanog is present in enriched pathways. Their row is marked by **

Column1	Category	Term ID	Term description	Observed gene count	Strength	False discovery rate
**1***	GO Function	GO:0005515	Protein binding	41	0.37	5.90E-08
**2***	GO Function	GO:0005488	Binding	50	0.21	6.74E-08
**3**	GO Function	GO:0005102	Signaling receptor binding	19	0.7	2.44E-06
**4**	GO Function	GO:0008083	Growth factor activity	8	1.36	3.46E-06
**5**	GO Function	GO:0008201	Heparin binding	7	1.25	0.00016
**6***	GO Function	GO:0003682	Chromatin binding	11	0.86	0.00021
**7**	GO Function	GO:0044877	Protein-containing complex binding	16	0.64	0.00023
**8**	GO Function	GO:0050839	Cell adhesion molecule binding	8	1.05	0.00037
**9**	GO Function	GO:0005104	Fibroblast growth factor receptor binding	4	1.81	0.00039
**10**	GO Function	GO:0005178	Integrin binding	6	1.22	0.00087
**1**	GO Component	GO:0032991	Protein-containing complex	35	0.43	2.93E-07
**2***	GO Component	GO:0000785	Chromatin	13	0.91	5.51E-06
**3***	GO Component	GO:0005694	Chromosome	15	0.68	0.00019
**4**	GO Component	GO:0005615	Extracellular space	17	0.61	0.00019
**5**	GO Component	GO:0009986	Cell surface	13	0.72	0.00026
**6**	GO Component	GO:0009897	External side of plasma membrane	9	0.88	0.00089
**7**	GO Component	GO:0031012	Extracellular matrix	9	0.87	0.0009
**8**	GO Component	GO:0005583	Fibrillar collagen trimer	3	2.04	0.0011
**9**	GO Component	GO:0098552	Side of membrane	10	0.77	0.0011

**10**	GO Component	GO:0005576	Extracellular region	18	0.48	0.0017
**1***	GO Process	GO:0010033	Response to organic substance	34	0.66	4.66E-13
**2***	GO Process	GO:0070887	Cellular response to chemical stimulus	32	0.69	5.68E-13
**3***	GO Process	GO:0051173	Positive regulation of nitrogen compound metabolic process	33	0.64	4.10E-12
**4***	GO Process	GO:0071310	Cellular response to organic substance	28	0.75	4.71E-12
**5***	GO Process	GO:0070848	Response to growth factor	17	1.15	6.10E-12
**6**	GO Process	GO:0030334	Regulation of cell migration	21	0.96	6.10E-12
**7***	GO Process	GO:0010604	Positive regulation of macromolecule metabolic process	34	0.6	6.10E-12
**8***	GO Process	GO:0071363	Cellular response to growth factor stimulus	16	1.15	2.25E-11
**9***	GO Process	GO:0009653	Anatomical structure morphogenesis	28	0.71	2.25E-11
**10***	GO Process	GO:0048522	Positive regulation of cellular process	40	0.47	2.25E-11

Supp. Table S3*. Top 10 enriched TISSUES, KEGG, and Wikipathways pathways based on False discovery rate. Nanog is present in enriched pathways that their row is marked by *. The last row of Wikipathways (11**) represents an enriched pathway that wasn't among the top 10*

**TISSUES, KEGG and Wikipathways**						
**Column1**	**Category**	**Term ID**	**Term description**	**Observed gene count**	**Strength**	**False discovery rate**
**1***	TISSUES	BTO:0002322	Cell property	10	1.97	1.25E-13
**2***	TISSUES	BTO:0006078	Pluripotent stem cell	10	1.51	1.00E-09
**3***	TISSUES	BTO:0000000	Tissues, cell types and enzyme sources	45	0.34	1.00E-09
**4***	TISSUES	BTO:0000669	Embryonic cell line	9	1.56	3.11E-09
**5***	TISSUES	BTO:0001489	Whole body	44	0.33	3.11E-09
**6**	TISSUES	BTO:0001486	Skeletal system	13	1.12	4.75E-09

**7**	TISSUES	BTO:0002050	Osteogenic cell	8	1.63	5.97E-09
**8***	TISSUES	BTO:0000284	Organism form	22	0.72	5.97E-09
**9**	TISSUES	BTO:0001593	Osteoblast	7	1.75	1.60E-08
**10***	TISSUES	BTO:0001099	Blastocyst	6	1.97	2.32E-08
**1**	KEGG	mmu05218	Melanoma	7	1.63	1.62E-07
**2**	KEGG	mmu05200	Pathways in cancer	12	1	3.78E-07
**3**	KEGG	mmu04151	PI3K-Akt signaling pathway	10	1.09	8.76E-07
**4**	KEGG	mmu04015	Rap1 signaling pathway	8	1.23	2.46E-06
**5***	KEGG	mmu05205	Proteoglycans in cancer	7	1.19	2.52E-05
**6**	KEGG	mmu05226	Gastric cancer	6	1.25	7.21E-05
**7**	KEGG	mmu05144	Malaria	4	1.53	0.00038
**8**	KEGG	mmu04810	Regulation of actin cytoskeleton	6	1.09	0.00041
**9**	KEGG	mmu04014	Ras signaling pathway	6	1.07	0.00051
**10***	KEGG	mmu04550	Signaling pathways regulating pluripotency of stem cells	5	1.2	0.00062
**1***	WikiPathways	WP1763	Mechanisms associated with pluripotency	11	1.22	1.15E-08
**2**	WikiPathways	WP2841	Focal adhesion: PI3K-Akt-mTOR signaling pathway	10	1.14	2.82E-07
**3**	WikiPathways	WP339	ESC pluripotency pathways	6	1.36	2.18E-05
**4**	WikiPathways	WP5242	Comprehensive IL-17A signaling	5	1.33	0.00021
**5**	WikiPathways	WP3632	Lung fibrosis	4	1.46	0.00055
**6**	WikiPathways	WP523	Regulation of actin cytoskeleton	5	1.16	0.00091
**7**	WikiPathways	WP458	Inflammatory response pathway	3	1.64	0.0017
**8**	WikiPathways	WP85	Focal adhesion	5	1.07	0.0018
**9**	WikiPathways	WP2432	Spinal cord injury	4	1.25	0.0019
**10**	WikiPathways	WP2375	miRNAs and TFs in iPS Cell Generation	2	2.1	0.0036
**11****	WikiPathways	WP723	Wnt signaling pathway and pluripotency	3	1.13	0.0206

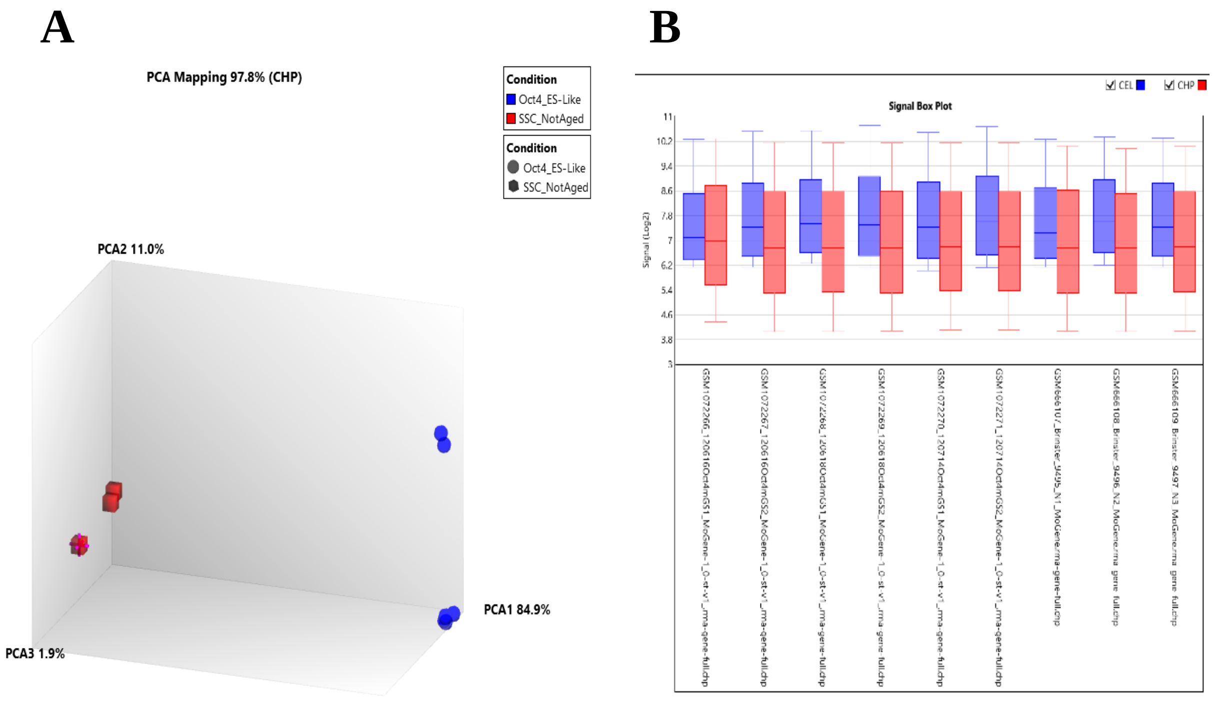

Supp. Figure S1. (A) The results of the principal component analysis (PCA), indicate that 97.8% of the variance is accounted for by the principal components. (B) Data normalization results of the microarray data.

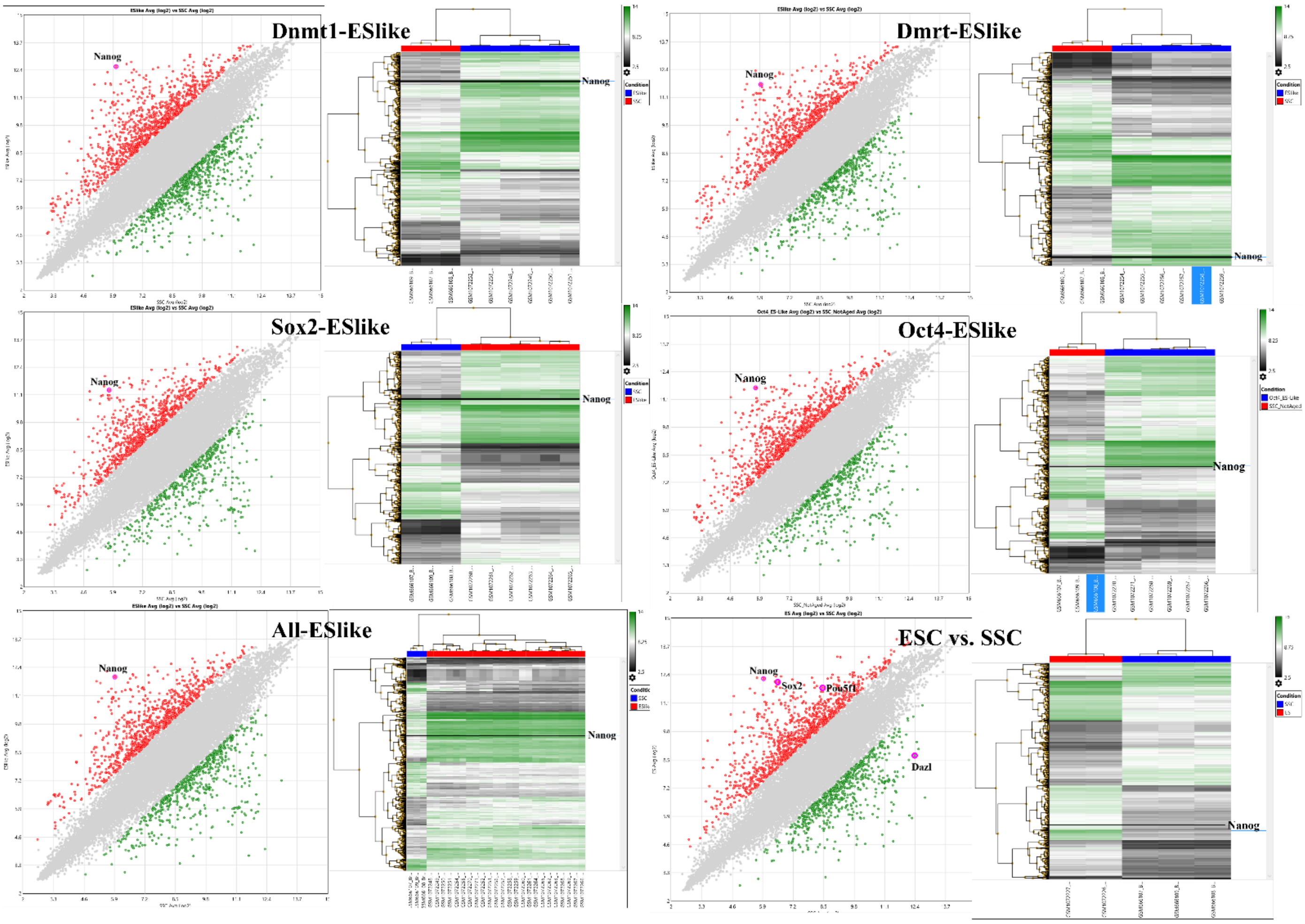

Supp. Figure S2. Confirmation analysis was conducted on samples using different combinations to test our main analysis. The results revealed consistent gene expression patterns across different combinations and samples. Specifically, Nanog and other crucial genes within the graphs exhibited similar patterns to those observed in the main figures of the analysis.

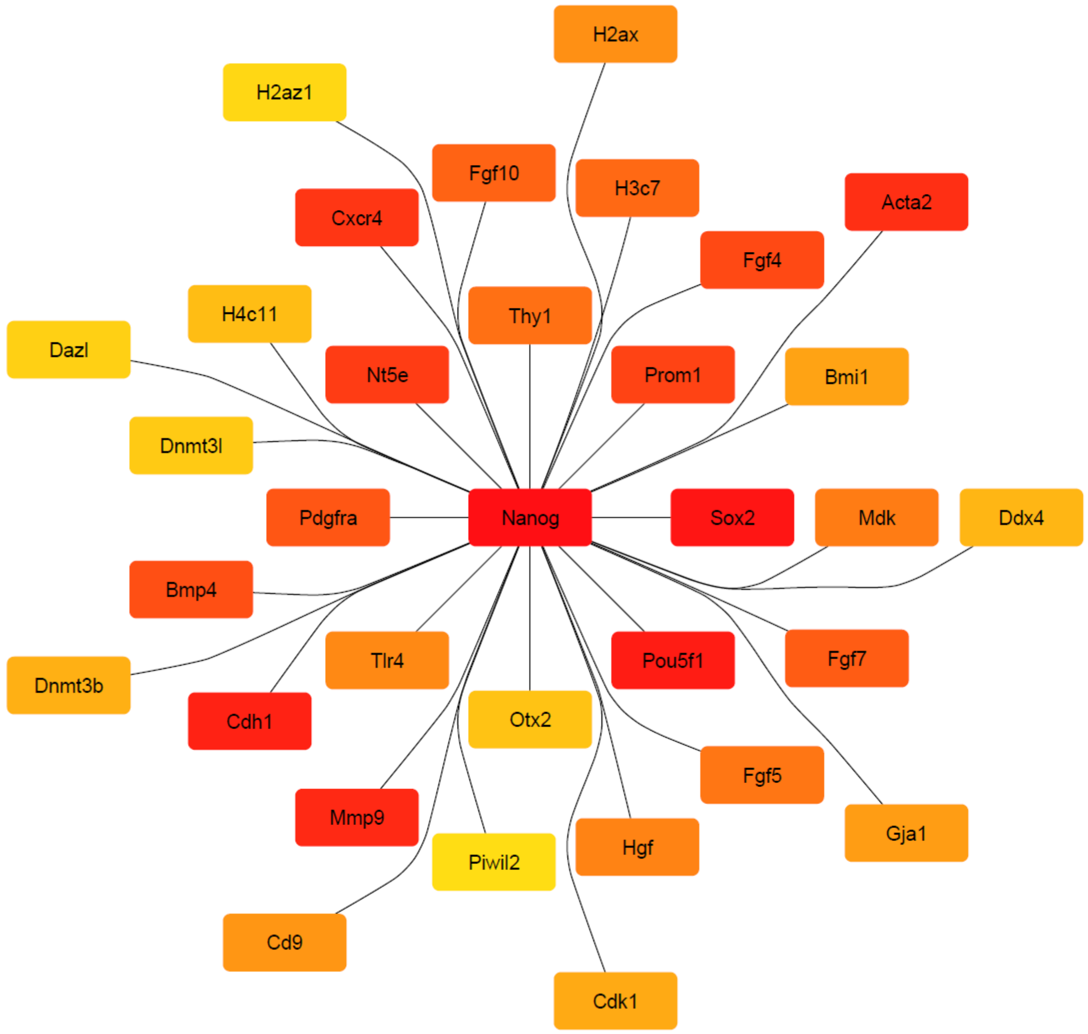

Name	Score
Nanog	8.879
Sox2	8.541
Pou5f1	8.535
Cdh1	8.008
Mmp9	7.775
Acta2	7.61
Cxcr4	7.551
Nt5e	7.372
Prom1	7.345
Fgf4	7.296
Bmp4	7.262
Pdgfra	7.259
Fgf7	7.24
Fgf10	7.226
H3c7	7.143
Thy1	7.102
Fgf5	7.023
Mdk	6.725
Hgf	6.54
Tlr4	5.869
H2ax	5.857
Cd9	5.743
Gja1	5.666
Bmi1	5.622
Cdk1	5.455
Dnmt3b	5.218
Ddx4	4.668
H4c11	4.46
Otx2	4.228
Dnmt3l	4.203
Dazl	3.663
H2az1	3.559
Piwil2	2.933

Supp. Figure S3. *The hub gene network of Nanog and its connected nodes are visualized by the EPC method of the Cytohubba app. Darker colors represent higher scores and as a result, higher importance and fainter colors have lower scores, which represents lower importance in the network.*
